# Are some patient-perceived migraine triggers simply early manifestations of the attack?

**DOI:** 10.1007/s00415-020-10344-1

**Published:** 2021-01-05

**Authors:** Nazia Karsan, Pyari Bose, Jayde Newman, Peter J. Goadsby

**Affiliations:** 1 Headache Group, Department of Basic and Clinical Neuroscience, Institute of Psychiatry, Psychology and Neuroscience, King’s College London, London, UK; 2 NIHR-Wellcome Trust King’s Clinical Research Facility, SLaM Biomedical Research Centre, King’s College Hospital, London, UK; 3 Present Address: Department of Neurology, Auckland City Hospital, Auckland, New Zealand

**Keywords:** Migraine, Premonitory, Prodrome, Triggers, Headache

## Abstract

**Objective:**

To study the agreement between self-reported trigger factors and early premonitory symptoms amongst a group of migraineurs in both spontaneous and pharmacologically provoked attacks.

**Methods:**

Fifty-three subjects with migraine with and without aura, with ≤ 22 headache days/month, with spontaneous premonitory symptoms associated with migraine attacks were recruited nationally. A detailed history was taken by a study investigator to confirm diagnosis and extended phenotyping was performed to identify patient-reported triggers for migraine attacks, premonitory symptom phenotype and headache characteristics, using a standardised physician-administered questionnaire. The same subjects were exposed to a 0.5 mcg/kg/min nitroglycerin infusion over 20 min, to determine if similar migraine symptoms could be triggered. The triggered attacks were phenotyped in the same way as spontaneous ones. Percentage agreement and Cohen’s kappa measure of agreement were used to identify concordance between patient-reported triggers and the corresponding spontaneous and triggered premonitory symptoms. Percentage agreement of > 60% and/or a kappa value > 0.3 with *P* < 0.05 were considered significant.

**Results:**

There was statistically significant agreement between perception of light as a migraine trigger and spontaneous premonitory photophobia; perception of sound as a trigger and triggered premonitory phonophobia; skipping meals as a trigger and spontaneous premonitory food cravings; and food triggers and spontaneous premonitory food cravings. There was good agreement between stress and premonitory triggered mood change.

**Conclusions:**

At least some patient-reported triggers, such as light, sound, foods and skipping meals, may represent early brain manifestations of the premonitory phase of the migraine attack.

## Introduction

Migraine is a common and disabling disorder [1]. Despite considerable therapeutics research over several decades, not all patients are well served by migraine treatments [2, 3]. This can leave patients and physicians frustrated and searching for other options for disease management. Many search for attack triggers as a preventive strategy and can make extensive dietary and lifestyle modifications to try to help their headache burden [4]. However, despite patient reports of a wide range of triggers in clinical practice, there is little objective evidence for commonly associated triggers, such as chocolate [5] and bright lights [6], reproducing migraine in experimental studies.

Increasingly recognised in migraine is the premonitory phase, the earliest phase of the attack, which is characterised by sensory sensitivities, food cravings and mood change, occurring hours to days prior to pain onset [7]. A range of prevalence of premonitory symptoms is reported in the literature, across varying study designs, with a general increase in prevalence reported with time [8–14], suggesting that perhaps increased attention to this phase, patient and physician education, and recognition of these symptoms have led to an increase in symptom identification. The lack of experimental evidence for many migraine triggers, and the generally poor response in migraine frequency to avoidance of such factors [15], has prompted a re-evaluation of whether patient-perceived migraine triggers are true triggers, or, in some part, incorrectly attributed early premonitory manifestations of the attack [16, 17]. It is feasible that if a patient experiences a symptom before migraine headache, such as a chocolate craving, they may then go and consume some chocolate, and then when they develop a headache some hours to a day later, assume that the chocolate triggered the headache, when in fact the migraine attack had likely already started within the brain.

Recently, functional neuroimaging studies have suggested that there is early engagement of various brain areas during the premonitory phase; namely the hypothalamus, thalamus, limbic areas, sensory processing cortex and brainstem regions [18–24]. These areas functionally correlate with commonly reported symptoms during this time, such as thalamus and sensory cortices with photophobia, hypothalamus with homeostatic dysregulation and altered arousal and limbic areas with mood and cognitive change. Alterations in homeostasis and arousal, sensory perception, and mood and cognition are reported as premonitory symptoms and triggers. Early brain changes mediating premonitory symptoms before headache may, therefore, lead to the correct association of these symptoms with corresponding trigger factors, but incorrect attribution that the trigger factor is directly causing the headache.

We wished to examine reported triggers and premonitory symptoms amongst the same individuals in patients with migraine with and without aura, to look for such an association in both retrospectively reported spontaneous attacks, and prospectively observed nitroglycerin-triggered attacks. The latter, in case subjects had not themselves noticed particular premonitory symptoms associated with spontaneous attacks. The range of prevalence of these symptoms reported in the literature suggest that under-recognition is likely to be an issue at least for some patients. Nitroglycerin (NTG) has been shown to be able to trigger both premonitory symptoms and migraine headache, thus making it a suitable experimental migraine model for this study [25]. In addition, the phenotype of premonitory symptoms following nitroglycerin has been shown to be comparable to those following spontaneous attacks in the same individual [26].

### Methods

The methodology used in this study has been previously reported [26] and is summarised here.

### Subject recruitment

Subjects with migraine were identified through online advertisements, bulletins, patient group advertising and university advertising and through local and national headache clinics. The inclusion criteria for the study included a diagnosis of migraine with or without aura, using ICHD-3 beta, which was in use at the time of the study [27]. We identified subjects with up to 22 headache days a month, a history of spontaneous premonitory symptoms with attacks, and no medical or psychiatric contraindications to study participation and/or NTG exposure. Use of any oral single agent oral preventive therapy for migraine was allowed. Exclusion criteria included medication overuse, illicit drug use, excess alcohol consumption and smoking. Use of more than one oral preventive agent for migraine, the use of neuromodulatory devices, or both, and onabotulinum toxin type A and/or greater occipital nerve injections within the past 3 months were also excluded.

Given the generally poor ability of NTG to trigger aura [28–30], we did not anticipate that aura would be triggered in the study and act as a potential confound to identification of premonitory symptoms. We included subjects on single agent preventive therapy to aid recruitment, noting that it has never been systematically studied if the use of migraine preventive agents alters the reporting of premonitory symptoms. Recruitment was completed from February 2015 to July 2017.

### Sample size

The initial nitroglycerin triggering study, from which these data are extracted, aimed to study sufficient subjects exposed to nitroglycerin to exceed our previous experience [25]. We, therefore, aimed to expose at least 50 subjects to nitroglycerin.

### Screening

Three hundred and fifty subjects were pre-screened for eligibility. Of these 350 subjects, 53 (15%) met eligibility criteria, agreed to attend a screening visit and were included in the final analysis. There was a large pre-screening failure rate, mostly due to too frequent headache and use of more than one migraine preventive agent.

All study visits were performed within the Clinical Research Facility at King’s College Hospital, London, UK.

**Fig. 1:**
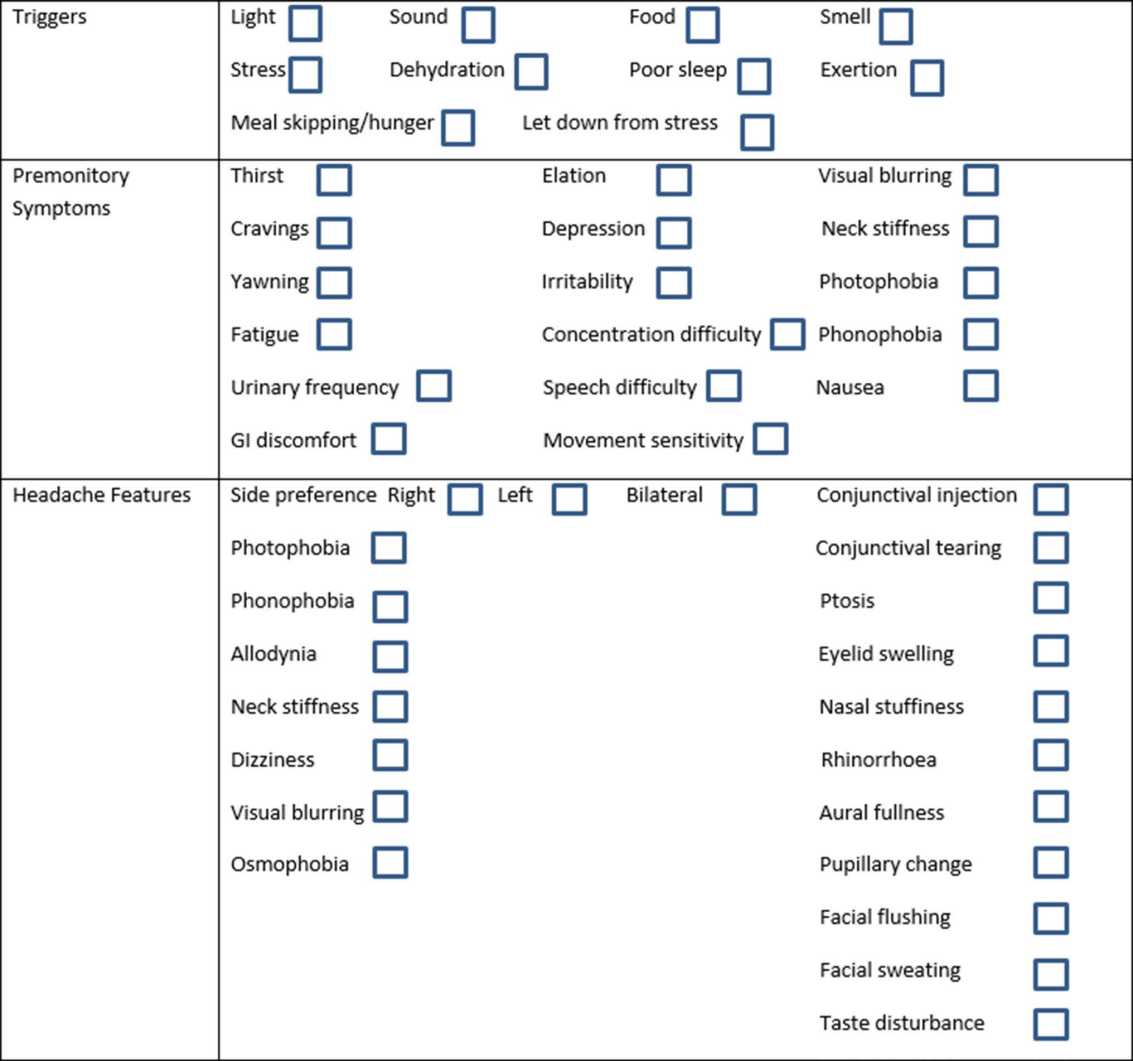
Phenotypic data collection questionnaire for both spontaneous and triggered attacks

A prior history of headache or any migraine symptoms or treatment was not allowed 12 h prior to the study visit. The screening visit involved written consent for study participation, followed by detailed phenotyping of spontaneous migraine attacks, triggers, medication history and ensuring no medical or pharmaceutical contraindications to any of the study drugs, including nitroglycerin and acute migraine treatments used to treat pain in the study: intravenous aspirin and subcutaneous sumatriptan. Each symptom or trigger was reported as a yes/no answer, and all positive responses were recorded, including the reporting of multiple triggers. An appropriate cardiovascular and neurological examination was performed to exclude obvious cardiac contraindications to study participation and secondary cause for a headache disorder. An ECG was performed to exclude cardiac contraindications to nitroglycerin or triptan exposure. The spontaneous migraine attacks were retrospectively phenotyped in detail, using a physician-administered symptom questionnaire (Fig. 1). Subjects were allowed to volunteer any triggers or symptoms not mentioned in the questionnaire.

### Nitroglycerin triggering

Following the history and examination, each subject was exposed to a 0.5-mcg/kg/min nitroglycerin infusion over 20 min. Subjects were symptomatically and haemodynamically assessed with blood pressure, heart rate, and oxygen saturation monitoring before the infusion and at 5-min intervals during the infusion. Each subject was prospectively questioned regarding the evolution of any headache, its site, severity, phenotype and the presence of any other symptoms, including typical premonitory symptoms using the same physician-administered symptom questionnaire that was used for spontaneous attacks (Fig. 1). Questioning continued at 15-min intervals following the infusion until the time of headache resolution following treatment. The answers to the premonitory symptom question presence were binary (yes/no), without any further grading of intensity or severity.

A premonitory symptom was defined as any symptom that the patient experienced following nitroglycerin infusion in the absence of migraine headache, which was typical for a symptom they would experience prior to a spontaneous migraine headache.

Nitroglycerin is a drug agent with vasodilatory actions and a short half-life (minutes) [31]. Within the brain, the vasodilatory drug effects are thought to peak at a similar time, and possibly last for no more than 45 min [32, 33]. Drug side effects typically come on quickly after intravenous administration and usually resolve within 5–10 min. We would, therefore, hope that with the design of the study, drug effects, if any, would be minimal or absent during premonitory symptoms.

Whilst our previous work has looked at symptoms triggered across serial nitroglycerin exposures and following placebo [26], for this study, we aimed to compare only spontaneous and nitroglycerin-triggered symptoms on the first nitroglycerin exposure.

### Treatment of triggered attacks

All delayed migrainous headache following nitroglycerin was treated as soon as it reached moderate-severe intensity, using either 1 g intravenous aspirin or 6 mg subcutaneous sumatriptan, based on subjects’ usual response. Headache freedom was necessary before a subject could be discharged from the Research Facility.

### Statistical analysis

Percentage agreement between spontaneous trigger factor and spontaneous and triggered reporting of the corresponding premonitory symptom and Cohen’s kappa analysis were used to assess agreement between selected trigger factors and corresponding premonitory symptoms. For subjects who reported more than one spontaneous trigger, each trigger factor was analysed for association with the corresponding premonitory symptom separately. The groups were selected based on feasibility of the triggers and premonitory symptoms being associated:

- Light trigger—Premonitory photophobia.
- Sound trigger—Premonitory phonophobia.
- Stress trigger—Premonitory mood change.
- Stress trigger-—Premonitory neck stiffness.
- Food trigger—Premonitory food cravings.
- Dehydration trigger—Premonitory thirst.
- Hunger/skipping meals trigger—Premonitory food cravings.
- Poor sleep trigger—Premonitory fatigue.

All of these associations were tested for both spontaneous and nitroglycerin-triggered attacks. SPSS v 24 was used for all statistical analyses. Percentage agreement was calculated using a 2 × 2 crosstabulation of yes/no results for trigger and premonitory symptom and taking a sum of the yes/yes and no/no responses. Percentage agreement of > 60% and/or a kappa value of > 0.3 (fair-good agreement) with *P* < 0.05 (kappa significantly different to 0) was considered significant, as using kappa alone can have its limitations when assessing agreement between dichotomous variables [34].

### Results

### Subject demographics

Of the 53 subjects, nine were males. Twenty-seven had migraine with aura (51%), 20 had migraine without aura (38%) and 6 had chronic migraine (11%). Sixteen (30%) were on single agent preventive therapy. The age range of subjects was 18–50 years (mean 36 years), with up to 22 headache days per month (median 8 days, range 1–22 days). Subject demographics are summarised in Table 1.

**Table 1:** Summary of subject demographics (*n* = 53). NSAID: non-steroidal anti-inflammatory drug

Age	18–50 years, mean 36 years
Sex	9 males, 44 females
Baseline headache days per month (range, median)	0–22 days, median 8 days
Baseline headache diagnosis	27 Episodic migraine with aura 20 Episodic migraine without aura 7 Chronic migraine
Preventive use (*n* = 16)	Beta-blocker-6; amitriptyline-3; topiramate-3; pizotifen-2, candesartan-2
Acute abortive treatment	Non-steroidal anti-inflammatory 11 Triptan 25 NSAID/triptan combination 2 Paracetamol/NSAID combination 10 Paracetamol/codeine combination 5
Baseline MIDAS score (range, median, IQR)	0–201, median 20 (IQR 12–42)

### Nitroglycerin triggering

Forty-four subjects (83%) developed delayed migrainous headache following the nitroglycerin infusion (range 20–278 min following nitroglycerin, median 107 min), and all but one (98%) had at least one typical premonitory symptom preceding the headache (range 4–155 min, median 23 min). Typical aura symptoms were triggered in 7 of the 53 subjects (13%). The aura phenotypes were visual aura (one was not followed by delayed migraine), hemisensory, hemimotor and Alice in Wonderland syndrome.

### Spontaneous triggers and spontaneously reported premonitory symptoms

The results for spontaneously reported triggers and spontaneous premonitory symptoms are shown in Table 2. The significant results are highlighted in bold.

**Table 2:** Agreement analysis between spontaneously reported triggers and corresponding premonitory symptoms

Subject-reported trigger factor	Corresponding premonitory symptom	Number reporting trigger factor (*n*)	Number reporting spontaneous premonitory symptom (*n*)	Percentage agreement spontaneous attacks (%)	Kappa spontaneous attacks	*P* value spontaneous attacks
Light	Photophobia	24	12	**75**	**0.3**	**0.03**
Sound	Phonophobia	12	7	29	0.05	0.7
Stress	Mood change	26	37	46	0	0.5
Stress	Neck stiffness	26	30	53	0.01	0.5
Food	Cravings	17	3	59	**0.4**	**0.004**
Dehydration	Thirst	10	18	22	0.06	0.7
Hunger/skipping meals	Cravings	7	17	29	**0.3**	**0.02**
Poor sleep	Fatigue	13	41	22	0	0.4

The most commonly reported trigger was stress (stress and let-down from stress) (*n* = 26), followed by light (*n* = 24) and certain foods (*n* = 17). The most common premonitory symptoms reported during spontaneous attacks were concentration difficulty (*n* = 44), fatigue (*n* = 41), mood change (*n* = 34) and neck stiffness (*n* = 28). Between 1 and 9 premonitory symptoms were reported during spontaneous attacks (median 5, IQR 4–7). There was good agreement for the reporting of light as a trigger and spontaneous premonitory photophobia, food as a trigger and premonitory cravings and hunger or skipping meals as a trigger and premonitory cravings.

### Spontaneous triggers and triggered premonitory symptoms reported

The results for spontaneously reported triggers and triggered premonitory symptoms are shown in Table 3. The significant results are highlighted in bold.

**Table 3:** Agreement analysis between spontaneously reported triggers and nitroglycerin-triggered premonitory symptoms

Subject-reported trigger factor	Corresponding NTG-triggered premonitory symptom	Number reporting trigger factor (*n*)	Number reporting NTG-triggered premonitory symptom (*n*)	Percentage agreement NTG-triggered attacks (%)	Kappa NTG-triggered attacks	*P* value NTG-triggered attacks
Light	Photophobia	24	35	**63**	**0.5**	**< 0.001**
Sound	Phonophobia	12	11	**64**	**0.5**	**< 0.001**
Stress	Mood change	26	14	**64**	0.17	0.18
Stress	Neck stiffness	26	32	50	0.02	0.9
Food	Cravings	17	3	33	0	1
Dehydration	Thirst	10	21	19	0	1
Hunger/skipping meals	Cravings	7	3	33	0.13	0.3
Poor sleep	Fatigue	13	14	23	0	0.7

The most common premonitory symptoms reported during nitroglycerin-triggered attacks were fatigue (*n* = 43), photophobia (*n* = 35), concentration difficulty (*n* = 34) and neck stiffness (*n* = 32). Between 0 and 9 premonitory symptoms were triggered with nitroglycerin (median 5, IQR 3–6). There was good agreement for the reporting of light as a trigger and nitroglycerin-triggered premonitory photophobia, sound as a trigger and nitroglycerin-triggered phonophobia and stress as a trigger and nitroglycerin-triggered premonitory mood change.

**Fig. 2:**
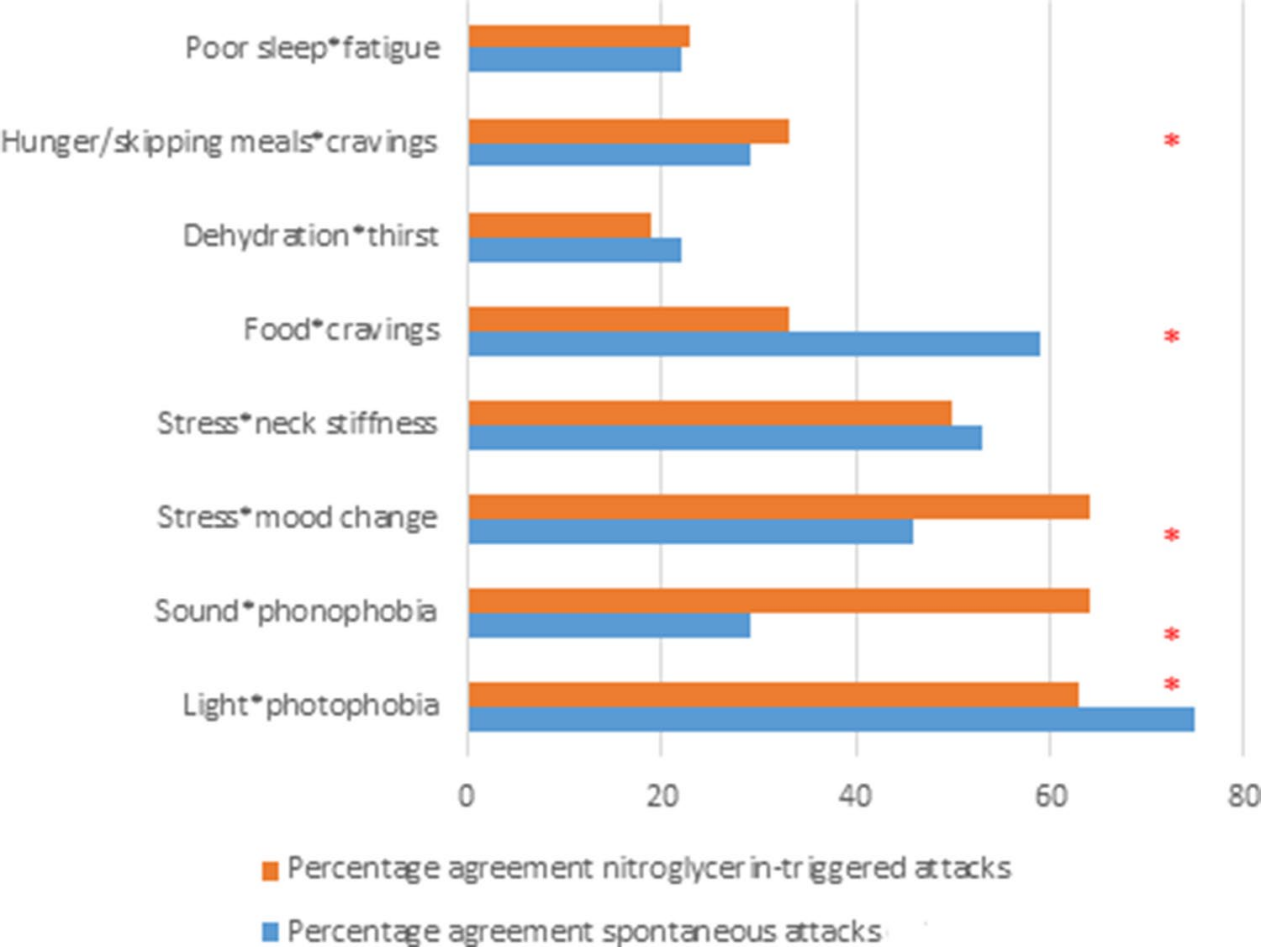
Summary of the percentage agreement between trigger factors and corresponding premonitory symptom in both spontaneous (blue) and nitroglycerin-triggered (orange) attacks [*Percentage agreement of > 60% and/or a kappa value of > 0.3 (fair-good agreement) with *P* < 0.05]

The frequency of reporting different trigger factors and associated premonitory symptoms during spontaneous (blue) and nitroglycerin-triggered (orange) migraine attacks are summarised in Fig. 2.

There are some differences in the associations between spontaneous and nitroglycerin-triggered attacks, which one could attribute to the environmental differences during spontaneous attacks and study visits, as well as increased patient recognition during direct observation of attacks and questioning on symptoms compared to retrospective recall of spontaneous attacks. This included increased recognition of symptoms like photophobia, phonophobia and movement sensitivity.

### Discussion

Our results show an association between some spontaneous patient-reported triggers, and spontaneous and nitroglycerin-triggered premonitory symptoms within the same subject. The findings are consistent with some reported triggers being premonitory symptoms associated with migraine that are mis-attributed by patients. Dissecting this relationship will help patients understand their own symptoms, and researchers build a better model of this complex neurological disorder.

During both spontaneous and triggered attacks, there was a significant association between the development of photophobia and light perceived as a trigger. There was discrepancy between spontaneous and triggered attacks for sound and phonophobia (triggered attacks only), stress and mood change (triggered attacks only), food and cravings and hunger and cravings (spontaneous attacks only). These differences are likely somewhat related to under-recognition of some premonitory symptoms by patients and, therefore, under-reporting in spontaneous attacks, as well as reporting bias caused by direct observation of triggered attacks. For example, it is feasible that mood change in the absence of a collateral witness may not be readily noticed by patients but could well have been noted by the physician during observation of triggered attacks. Similarly, food cravings were much less reported with triggered attacks than spontaneously, likely owing to environmental factors and lack of appetite stimulation during the triggered visit.

Whilst we have previously shown a comparable phenotype of spontaneous and nitroglycerin-triggered migraine attacks [26], it is clear that for some symptoms, there is a discrepancy, namely photophobia being more commonly triggered than reported spontaneously, and mood change being much more commonly reported spontaneously than during triggered attacks. In addition, the timeline of a nitroglycerin-triggered migraine attack is much shorter and generally compressed relative to a spontaneous attack. Triggered premonitory symptoms are typically followed by headache within 2 h [26], which is substantially less than what is seen in spontaneous attacks.

### Limitations and scope for further work

Clearly the relationship between what may be a perceived trigger for a migraine attack and certain premonitory symptoms is complex and unlikely to be unidirectional. Migraine is a disorder influenced by genetic and epigenetic factors. Migraine is prone to change depending on both endogenous (such as hormonal change) and exogenous factors (such as sleep, alcohol and stress levels) [35]. We did not, therefore, expect to find an association between all the triggers that we captured and their corresponding premonitory symptoms. In addition, the use of different methods of data collection (retrospective and prospective), and the environment associated with the triggering visits, may have impacted on premonitory symptom phenotype. Going forwards, use of electronic diary systems and device application technology, following patient education, to allow prospective recording of perceived triggers, premonitory symptoms and their association to headache during spontaneous attacks, would be a valuable means of assessing these relationships, understanding better the mechanisms behind attack initiation and advancing therapeutics. Either way, increased patient education and recognition of the heterogeneity of migraine and the systematic recording of factors such as perceived triggers and early painless symptoms is likely to improve our understanding of the disorder. Vital questions behind how and why a migraine attack starts are being advanced by neuroimaging such that pre-pain phase treatments are a reasonable prospect in the future. In an era where despite the emergence of novel and targeted migraine therapies, there remains a need for effective attack abortion for many sufferers, such an understanding is likely to contribute to novel advances.

It is also important to highlight that whilst we compared triggers and premonitory symptoms in spontaneous and nitroglycerin-triggered attacks, prospective observation of triggered attacks is arguably not the same as prospective observation of spontaneous attacks, which would be ideal. As discussed above, the differences in symptom capture and, therefore, association with triggers may have been in some part impacted on by nitroglycerin itself, or the environment in which it was administered, or indeed by the reporting bias induced by direct observation and questioning by a physician. Examining both spontaneous and triggered attacks in this subject cohort offered a reasonable starting point. Whilst we do not consider that there are likely to be fundamental biological differences between pharmacologically -provoked and spontaneously occurring migraine attacks (akin to alcohol-induced migraine attacks), the drug itself, the study environment and the recording of triggered symptoms may have impacted on the results and caused the observed differences in premonitory symptom association with triggers between spontaneous and triggered attacks. Spontaneous attacks occur in patients’ own surroundings (home, work, school) and most often occur in the absence of an exogenous trigger. Patients self-report their symptoms, whilst nitroglycerin-triggered attacks are provoked, are directly observed within a clinical facility and the phenotype of symptoms reported thereafter is captured both through patient self-reporting and through direct questioning by a physician. These differences are important to recognise, and highlight the importance of large-scale population-based prospective diary studies going forwards, to further inform on these relationships in the future.

## Conclusions

Despite the limitations discussed, there does seem to be a relationship between some triggers and premonitory symptoms. Such a misperception would be consistent with the generally poor outcomes of trigger studies involving putative agents, such as chocolate and light, and with premonitory phase biology driving behaviours that are correctly associated with the trigger factors yet falsely attributed to them. Emerging functional imaging work, as well as animal migraine models looking at some premonitory symptoms, has suggested that early activation of brain areas, pathways and neuropeptide systems occur during the premonitory phase and are responsible for mediating some of these symptoms via hypothalamic and other diencephalic mechanisms [36]. There is, therefore, a neuroimaging correlate for how these symptoms may be mediated centrally, for example, with the thalamus and connections to visual cortex likely being involved in photophobia and hypothalamic connections to pontine areas and pontine connections to cortical areas being involved in regulation of feeding [24].

Going forwards, systematic and prospective attack recording is likely to be useful in advancing neuroimaging work and therapeutics.

## Data Availability

The principal author had full access to those data and has maintained the right to publish any and all data independent of any third party. The data (anonymised) and full study protocol are available at request.
